# Eating behaviour patterns in Chinese children aged 12-18 months and association with relative weight - factorial validation of the Children's Eating Behaviour Questionnaire

**DOI:** 10.1186/1479-5868-9-5

**Published:** 2012-01-24

**Authors:** Ying-Ting Cao, Viktoria Svensson, Claude Marcus, Jing Zhang, Jian-Duan Zhang, Tanja Sobko

**Affiliations:** 1 Karolinska Institutet, Department of Clinical Science, Intervention and Technology, Division of Pediatrics, Karolinska University Hospital Huddinge, SE 141 86 Stockholm, Sweden; 2 Tongji Medical College School of Public Health, Huazhong University of Science and Technology, 13 Hangkong RD, Wuhan, 430030, P.R.China

**Keywords:** Eating behaviour, CEBQ, Chinese overweight children, obesity, factorial validation

## Abstract

**Background:**

Eating behaviours have been suggested relating to obesity development. The Children's Eating Behaviour Questionnaire (CEBQ) is a parent-report measure constructed to assess multiple dimensions of eating behavior for children. This study aimed to test the validity of the Chinese version of Children's Eating Behaviour Questionnaire (CEBQ) in Chinese children aged 12-18 months. We examined factor structure and the reliability of the Chinese version of the CEBQ, the associations between children's eating behaviours and children's weight (BMI SDS) were assessed.

**Methods:**

219 questionnaires were filled out by the caregivers, approached in community health care centers in two cities in China. BMI of each child was calculated and converted to BMI SDS. Factor validation (Principal Component Analysis, exploratory factor analysis) on all CEBQ items was performed and gender difference in eating behaviours was examined. Correlations between eating behaviours and the child's BMI SDS were analyzed by linear regression analysis controlling for gender, parental combined weight, and education.

**Results:**

The factor analysis revealed a seven-factor solution, with factor 'food responsiveness' (FR) split into two. 'Satiety responsiveness' (SR) and 'Enjoyment of food' (EF) factors were not detected. Interestingly, boys scored higher than girls in the FR scales, whereas girls had a higher score in 'food fussiness' (FF) scale.

**Conclusions:**

We conclude that although a valuable psychometric instrument, CEBQ might be affected by age and cultural differences. Therefore, adjusting it in order to fit the Chinese population was suggested. We did not find an association between eating behaviours and children's BMI SDS, when it was controlled for gender and parental weight.

## Background

Childhood obesity has become one of the most serious public health challenges worldwide in the last century, with 42 million overweight children under the age of five. The vast majority (80%) of these live in low and middle-income countries [1]. This trend has affected China and the prevalence of childhood obesity in some urban Chinese populations is already reached similar levels of the developed countries [2]. In 2005, total 7.73% of Chinese youth were overweight and 3.71% of them were obese. Partly explained by the explosive economic development and improvement of living conditions in China [3], general raise in income in China today enables a decreasing consumption of cereals and starchy roots, and an increasing consumption of high-caloric foods [4]. In addition, fast food and snack consumption together with increased TV screen time has been suggested to have contributed to the increase of childhood obesity prevalence in China [5].

Different patterns of eating behaviours have been suggested to influence the weight gain. For example, Schachter [6] showed that responsiveness to satiety, low in obese individuals, leads to failure to regulate their energy intake and to overeating. Furthermore, speed of eating was indicated as important factor for adiposity development [7,8].

A few standardized psychometric tools to measure children's eating behaviours have been developed. Some examples of these are the Dutch Eating Behaviour Questionnaire (DEBQ) [9], the Children's Eating Behaviour Inventory (CEBI) [10] and Children's Eating Behaviour Questionnaire (CEBQ) [11]. The latter is regarded as the most comprehensive instrument to assess children's eating behaviour and it has been validated with high reliability and validity in several countries [11-14] and recently even in Sweden [15]. It describes children's eating behaviour from eight dimensions. Satiety responsiveness (SR) assesses the sensitivity to the internal food cues or the threshold in each individual when deciding to stop eating. Overweight children are prone to eat more and fail to regulate their food intake, leading to overeating and obesity, compared with normal weight children [16]. The Enjoyment of food (EF) and Food responsiveness (FR) both assess general food preference in children as well as the desire to eat [12]. The former assesses the general appetite and the latter assesses the level of appetite and the intention of eating under external cues [12]. Drink desire (DD) is about preferring sweet drinks, mostly without thirst or hunger [17]. Slowness in eating (SE) and Food fussiness (FF) reflect low interest and enjoyment of food. Studies on speed of eating have shown that obese children complete a meal faster than the lean children [7,8]. Fussy eaters are inclined to eat less and slower [18-20] leading to a slower weight gain [20,21]. At the same time, regarding the relationship between fussy eating and weight, no difference was found between normal weight and obese children [20]. Emotional overeating (EOE) and Emotional under eating (EUE) refer to the increase or decrease in eating under negative emotions, involving stress [22]. According to 'psychosomatic theory' [23], EOE under negative emotion could lead to excessive weight gain.

CEBQ has been validated for both Dutch and Portuguese populations [13,14]. A similar Swedish study validated the CEBQ among a Swedish young population which belongs to a big project on obesity prevention and intervention focus on diet, sleep and physical activities in Sweden [24]. Part of this project is conducted in China, and we therefore intended to validate the CEBQ in a Chinese context.

The present study aims to examine the factor structure of the original CEBQ and investigates its applicability in Chinese culture background. We also hypothesized that eating behaviours is could be associated with young children's relative weight.

## Methods

### Procedures and participants

The CEBQ was translated into Chinese (Mandarin), read and corrected by several Chinese native speakers. The Chinese version was then translated back to English, thereafter adjusted and translated again to Chinese in order to ensure the congruence between the two different languages.

A total of 219 children with 114 boys and 105 girls, aged from 12 to 18 months from two Chinese cities (Zhenjiang and Wuhan) participated in this study. Children within this age range and without physical or psychological problems were recruited from three local community health centers in Zhenjiang and Wuhan respectively, after parents' understanding and signing the informed consent form. Children's weight and height were measured on-spot at the community health care centers, using a calibrated infant length and weight scale (WSH-I, Wuhan computer software development Co. Ltd.). At the same occasion, all 219 questionnaires were completed by 184 parents and 35 grandparents with the assistance of study investigators when necessary. Each questionnaire was filled out only by one parent or grandparent, the person who took care of the child in the daily life. The demographic and anthropometric characteristics of the sample, as well as parental educational level were assessed (Table 1). Chinese percentiles and SD curve for boys and girls aged from 0-18 years old [25] were adopted as cutoff of overweight and obesity for children. The threshold for parents was according to Chinese adult BMI reference [26] with 24 ≤ BMI < 28 regarded as overweight, and BMI ≥ 28 regarded as obesity.

**Table 1:** Demographic and BMI characteristics of the sample (n = 219)

	Mean (SD), Range	N (%)
Children's BMI SDS	0.66 (1.15), (-3.7-5.3)	
Children's weight categories(a)	BMI	
Underweight (< 5^th^)	12.5	1 (0.5)
Normal weight (5^th ^≤ BMI < 85^th^)	17.2	111 (50.7)
Overweight (85^th ^≤ BMI < 95^th^)	18.6	48 (21.9)
Obese (95^th ^≤ BMI)	20.5	59 (26.9)
Parental BMI (kg/m^2^)		
Mother	21.1 (2.6), (16.6-32.0)	
Father	23.0 (2.9), (16.5-33.9)	
Combined parental weight		
No overweight or obese parent	128 (58)	
At least one overweight or obese parent	91 (42)	
Parental weight categories (b)		Mother/Father
Underweight (BMI < 18)		16/7 (7.3/3.2)
Normal weight (18 ≤ BMI < 24)		178/137 (81.2/62.6)
Overweight (24 ≤ BMI < 28)		20/64 (9.1/29.2)
Obese (28 ≤ BMI)		5/11 (2.3/5)
Parental education		
University and college		145/159 (66.2/72.6)
High school		68/57 (31.1/26)
Elementary school		6/3 (2/7/1.4)

^a ^Chinese children's reference (Li H, Ji CY, Zong XN, & Zhang YQ, 2009);

^b^Chinese adults' BMI criteria (Ji CY,2005);

### Data processing

The CEBQ consists of 35 items in the form of eight sub-scales, with each sub-scale loaded by 3 - 6 items. The items are rated on a five-point Likert scales, from 1 - 5 (1 = never, 2 = seldom, 3 = sometimes, 4 = often, 5 = always).

Body mass index (BMI) was calculated for parents and children respectively; children's BMI percentile was calculated according to Chinese latest reference. BMI for the child was adjusted for age and gender according to an international standard, BMI SDS [27]. Parents were divided into two groups given their calculated BMI. One group contained no overweight or obese parents; the other group contained at least one overweight or obese parent.

### Statistical procedures

All statistical analyses were conducted in STATISTICA 9.0 (data analysis software system, version 10. StatSoft, Inc. http://www.statsoft.com). In order to understand the underlying structure of the Chinese translation and ascertain whether it is similar to the original scale, exploratory factor analysis (EFA) was performed by using Varimax rotation on all items of the CEBQ. In order to allow factor inter-correlated, we used SPSS program to run this oblique rotation (Oblimin direct). Since the original CEBQ had an eight-factor structure, the number of factors was equally set to eight. Threshold for factor loadings was set as 0.6. Factors' reliability was assessed by using Cronbach's alpha coefficient. 0.7 was considered as general Cronbach's alpha cutoff, but the value could be lower for research compared to clinical situation [28]. Apart from 0.6 we even tried loading with 0.4 for factor analysis.

Score differences in factors between boys and girls and parental combined weight groups were tested by the independent t-test. The correlation between sub-scales of the CEBQ was analyzed by Pearson correlation.

Finally, linear regression was used to test the association between children's BMI SDS (dependent variable) and each eating behaviour scale, controlling for gender, parental combined weight and parental educational level. We additionally checked a simple correlation between child BMI SDS and the CEBQ scales in order to avoid the possible attenuating effect of parental weight and other confounding factors.

## Results

### Structure of translated scale and reliability

The factor analysis revealed a seven-factor solution (SE, EUE, FF, FR 1, DD, EOE, FR 2) with factor FR split into two (FR 1, FR 2). Variability among all 35 items was 52.1%, in terms of the seven factors (Table 2). Items belonging to each factor in Table 2 were loaded above 0.6. However, we omitted one item (number 5) in factor 1 (see table 2 and appendices) even though it loaded larger than 0.6. Because this item originally belonged to factor EF (enjoyment of food), rather than the other three homogenous items (all belong to factor SE (slowness of eating)), so it was excluded in factor SE.

**Table 2:** Factor loadings on Varimax Rotated Solution of Principal Components analysis (CEBQ, N = 219)

Scale name and items	Loading	Scale name and items	Loading
**Slowness in eating (Factor 1: 11.7% variance)**		**Food responsiveness 1 (Factor 4: 6.2% variance)**	
4. My child finishes his/her meal quickly	0.66	28. Even if my child is full up s/he finds room to eat his/her	0.67
8. My child eats slowly	0.76	favourite food	
18. My child takes more than 30 minutes to finish a meal	0.63	34. If given the chance, my child would always have food in his/her mouth	0.68
**Emotional under-eating (Factor 2: 6.0% variance)**			
11. My child eats less when s/he is tired	0.62	**Drink desire (Factor 5: 7.1%variance)**	
23. My child eats more when she is happy	0.60	6. My child is always asking for a drink	0.80
25.My child eats less when upset	0.76	29. If given the chance, my child would drink continuously throughout the day	0.80
**Food fussiness (Factor 3: 6.8% variance)**		31. If given the chance, my child would always be having a drink	0.82
7. My child refuses new food at first	0.71	**Emotional overeating (Factor 6: 6.6%variance)**	
10. My child enjoys tasting new foods	0.77	2. My child eats more when worried	0.65
32. My child is interested in tasting food s/he hasn't tasted before	0.75	13. My child eats more when annoyed	0.74
		15. My child eats more when anxious	0.77
		**Food responsiveness 2 (Factor 7: 7.7% variance)**	
		12. My child is always asking for food	0.65
		19. Given the choice, my child would eat most of the time	0.64

Factor structure was not affected by running Oblimin rotation in SPSS program compared with the results using Varimax rotation in STATISTICA program. It was neither affected by setting 0.4 as loading for factor analysis.

Internal consistency indicated by the Cronbach's alpha coefficient ranged from 0.52 to 0.80 for the CEBQ sub-scales (Table 3).

**Table 3:** Factor reliability of the CEBQ (N = 219)

	Cronbach's alpha	Average item-total correlation (range)
Slowness in eating	0.71	0.48(0.43-0.66)
Emotional undereating	0.60	0.33(0.30-0.46)
Food fussiness	0.71	0.53(0.49-0.57)
Food responsiveness 1*	0.52	0.35(0.35-0.35)
Desire to drink	0.80	0.65(0.59-0.68)
Emotional overeating	0.69	0.50(0.44-0.55)
Food responsiveness 2**	0.55	0.38(0,38-0.38)

* contains item 28 and 34; **contains item 12 and 19; item numbers refer to table 1

### Gender and combined parental weight difference

Significant gender differences in both food responsiveness sub-scales (FR 1 and FR 2) were identified (Table 4); boys scored higher than girls on FR 1 (mean 2.42 (SD 0.83) versus 2.08 (SD 1.01), p = 0.01) and FR 2 (mean 3.34 (0.83) versus 3.00 (1.04), p = 0.01). When it comes to Food fussiness (FF), boys scored significantly lower than girls (2.30 (0.84) VS 2.57 (0.87), p = 0.02). No significant difference in factors was found by combined parental weight.

**Table 4:** Gender difference in eating behavious (sub-scales)

Factors	Boy (N = 114)	Girl (N = 105)	P
			
	Mean (SD)	Mean (SD)	
Slowness in eating	2.90 (0.65)	2.96 (0.61)	0.43
Emotional undereating	3.09 (0.90)	2.95 (0.87)	0.23
Food fussiness	2.30 (0.84)	2.57 (0.87)	0.02*
Food responsiveness 1	2.42 (0.83)	2.08 (1.01)	0.01*
Desire to drink	3.14 (0.86)	2.96 (0.99)	0.14
Emotional overeating	1.82 (0.68)	1.75 (0.64)	0.46
Food responsiveness 2	3.34 (0.83)	3.00 (1.04)	0.01*

FR 1 = item 28, 34; FR 2 = item12, 19. *p < 0.05

Correlations between sub-scales of CEBQ and associations between children's weight and their eating behaviours

Our study indicated positive inter-correlations in the four 'food-approach' scales (FR 1, DD, EOE, FR 2) and the two 'food-avoidant' scales (SE, FF). Significant negative correlations (p < 0.05) were found between the two groups of scales (FF vs DD, r = -0.16, and FF vs FR 2, r = -0.24,). Sub-scales EOE and EUE (r = 0.21) were positively correlated (p < 0.05), in accordance with the previous studies. The correlations between sub-scales of CEBQ are shown in Table 5.

**Table 5:** Correlations between CEBQ sub-scales

	SE^a^	EUE^b^	FF^c^	FR^d ^1	DD^e^	EOE^f^	FR 2
SE	-	-	-	-	-	-	-

EUE	0.04	-	-	-	-	-	-

FF	0.26*	0.05	-	-	-	-	-

FR 1	0.07	-0.05	-0.06	-	-	-	-

DD	-0.06	0.18	-0.16*	0.16*	-	-	-

EOE	-0.05	0.21*	0.05	0.16*	0.21*	-	-

FR 2	0.02	0.10	-0.24*	0.24*	0.28*	0.13	-

*p < 0.05

^a ^Slowness in eating, ^b ^Emotional undereating, ^c ^Food fussiness, ^d ^Food responsiveness, ^e ^Desire to drink, ^f ^Emotional overeating.

There were no associations between child's BMI SDS and eating behaviours when controlled for gender, parental combined weight and parental educational level. We did not find any associations either between child's BMI SDS and eating behaviours (the factors revealed by factor analysis in this study), when we performed a simple correlation.

## Discussion

To our knowledge, this is the first time the CEBQ is tested in China. Also, this study is the first attempt to assess eating behaviour pattern among young Chinese children. We examined whether eating behaviours measured by the CEBQ are associated with children's weight in a sample of Chinese children at the age of 12-18 months.

Compared with the eight-factor structure in the original study [11], our study revealed seven CEBQ sub-scales (with two separate FR factors), and two factors could not be detected in our sample (EF and SR). Each sub-scale contained the items originally belonging to it, with an exception of the sub-scale factor SE, which in our study also had items originally belonging to another sub-scale (EF), such as item 5 (my child is interested in food).

Surprisingly, the original FR factor was split into two factors, each having two items loaded onto it, called FR 1 and FR 2 respectively. The items loaded onto FR 1 were 'Even if my child is full up, s/he finds room to eat his/her favourite food' and 'If given the chance, my child would always have food in his/her mouth'. The items loaded onto FR 2 were 'My child is always asking for food' and 'Given the choice, my child would eat most of the time'. However, one possible reason for two FRs could be the interpretation for 'always have food in the mouth'. Younger children are prone to keep the food in the mouth, and not swallowing immediately compared to older children.

We only show 19 items in table 2, although the original CEBQ scale has 35 items. The other missing16 items either did not load on any factors (loadings < 0.6) or were excluded even though loadings > 0.6 (item 5), or because they originally belonged to the two missing factors (EF and SR). The comparison of factor structure of this study with the original scale structure was indicated in additional file 1. When we lowered the loading from 0.6 to 0.4, it brought more items loaded on each factor. However, it didn't change the final factor results.

Regarding the two missing sub-scales, compared to the original factor structure of the CEBQ (EF and SR), they might not be applicable for the early age of the Chinese study group. The impact of the Chinese history and culture may also be taken into account of. The concept that 'a chubby baby is a healthy baby' is still presents today in spite of the growing awareness of childhood obesity in China. The one-child policy in China in the 1980s aggravated the situation when the only child became the cosset in the family. Moreover, in China, many grandparents take care of their only grandchild in an excessive way, traditionally reflecting in overfeeding practices. As a result, they provide their grandchildren with high energy-dense foods in far greater than needed amounts, increasing the potential risk for adiposity [29]. Consequently, young children could be tired of the food from an early age since they are always overfed, which could explain the missing EF and SR sub-scales.

Cronbach's alpha coefficient normally ranges between 0 and 1 but actually no lower limit to the coefficient is existed and the closer it is to 1, the stronger consistency the items in the scale [30]. In our study, low scores of the two FRs could be due to the inapplicability among particularly young children.

An interesting finding of this study is a significant gender difference in the FF and the two FR factors, suggesting that boys might be more interested in food than girls and girls might be more 'picky' than boys at an early age. Contradictory gender results in picky eating have been reported so far: either no gender differences [31,32] or boys scoring higher of food fussiness than girls [33]. However, another study has just recently reported the gender difference of 'food responsiveness' when using the Baby Eating Behaviour Questionnaire: male infants were slightly more responsive to food than female infants [34]. The issue is complicated by many aspects, for example, age. Addessi and others showed that this behaviour peaks at the age of 2 - 6, decreasing later all the way through adolescence [35].

In our study sample we found an unusually high proportion of overweight and obesity being almost half of the sample size. Although this should be checked in a bigger population, we still would like to report this. As mentioned above, this could be due to a common feeding practice in China today together with the lack of physical activity since small children are held by parents most of the time. No significant associations between children's relative weight (BMI SDS) and eating behaviour were observed in the present study. We also considered if adjusting parental weight could affect the correlation between children's BMI SDS and CEBQ scales. Therefore we also checked the simple correlation without controlling anything. The result remains the same and we therefore suggest that this could probably be explained by the young age of children in this study, as some eating behaviours are harder to detect at early age.

Moreover, different eating behavior in Chinese and European children might also contribute to this negative correlation, because CEBQ was developed on the basis of European children's eating behavior. This was also one main intention to conduct our study.

This study has several limitations. First, the sample population might not be representative for the whole Chinese children at the age of 12-18 months as it was tested only in two cites. Secondly, the parental weight and height were self-reported. In addition, the original CEBQ used oblimin rotation in factor analysis, we used varimax rotation instead, though no difference presented.

Despite of the drawbacks, this investigation pioneered a new field of studying young children's eating behaviours by using CEBQ in a totally different setting (China) and in younger children compared to the original and other studies. Based on our results we suggest certain adjustments in the Chinese version of CEBQ, such as to exclude the questions belonging to EF and SR scales, as they might be irrelevant for such an early age in Chinese children. In addition, our important observations regarding children's gender difference in eating behaviour such as FF and two FRs could have a further study.

## Conclusions

This study validated the CEBQ in the Chinese context and explored Chinese children's eating behaviour and its relationship with obesity in an early age. The Chinese version of CEBQ needs to be adjusted according to the Chinese situation, both because of the younger age, and also due to the different eating behavior in Chinese and European children. However, it offers a great opportunity to study eating behaviour in a different population and compare it with other studies.

## List of abbreviations

CEBQ: Children's Eating Behaviour Questionnaire; EF: Enjoyment of food; FR: Food responsiveness; EOE: Emotional overeating; DD: Desire to drink; SR: Satiety responsiveness; SE: Slowness in eating; FF: Food fussiness; EFA: Exploratory factor analysis; BMI SDS: Body mass index standard deviation score

## Competing interests

The authors declare that they have no competing interests.

## Authors' contributions

All authors were involved in all parts of this study, and they all read and approved the final manuscript. The contributions are described below.

YT C collected all data, performed the statistical analyses and wrote the whole manuscript. TS was responsible for the design of the study, and supervised the whole process. VS reviewed the manuscript and helped with the statistical analyses. JZh and JD Zh organized data collection in China and reviewed the manuscript. CM has given final approval of the version to be submitted.

## Supplementary Material

Additional file 1 **Comparison of the scale structure of this study with Original CEBQ**. There are only 19 items determined by this study, however there were 35 items originally developed in CEBQ. In this table, all 35 items are shown, including those items that were not found in this study but were originally developed in CEBQ to compare the structure of original study with this study. Click here for file
