# Supplementary material for: Eating behaviour patterns in Chinese children aged 12-18 months and association with relative weight - factorial validation of the Children's Eating Behaviour Questionnaire
Source: Int J Behav Nutr Phys Act. 2012 Jan 24;9:5. doi: 10.1186/1479-5868-9-5 (PMC3311563; doi:10.1186/1479-5868-9-5)
Supplement: Additional file 1 — Comparison of the scale structure of this study with Original CEBQ. There are only 19 items determined by this study, however there were 35 items originally developed in CEBQ. In this table, all 35 items are shown, including those items that were not found in this study but were originally developed in CEBQ to compare the structure of original study with this study. [file 1479-5868-9-5-S1.RTF]

Additional file 1
Scale name and itmes                                                    		Scale name and itmes		
Slowness in eating 　¡　¡　¡　¡　¡　¡　¡　¡　¡　¡　¡　¡　¡　¡　¡　¡　¡　¡　¡　¡　¡　¡　¡　¡　¡　¡　¡　¡　¡　¡　¡　¡　¡　¡　¡　¡　¡　¡　¡　¡　¡　¡　¡　¡　¡　¡　¡　¡　¡　¡　¡　¡　¡　¡　¡　¡　¡　¡　¡　¡　¡　¡　¡　¡　¡　¡　¡　¡　¡
4.My child finishes his/her meal quickly (both this and original study)　¡　¡　¡　¡　¡　¡　¡　¡　¡　¡　¡　¡　¡　¡　¡　¡　¡　¡ 
8.My child eats slowly (both this and original study)
18.My child takes more than 30 minutes to finish a meal (both this and original study)                          
35.My child eats more and more slowly during the course of a meal (original )
Emotional under-eating 
11.My child eats less when s/he is tired (both this and original study)                                                              
23.My child eats more when she is happy (both this and original study)                                                            
25.My child eats less when upset (both this and original study)                                        
9.My child eats less when angry (original)                           
Food fussiness (Factor3: 6.8% variance)
7.My child refuses new food at first (both this and original study)                                                                
10.My child enjoys tasting new foods (both this and original study)                                                                    
32.My child is interested in tasting food s/he hasn't tasted before (both this and original study)                                              
16.My child enjoys a wide variety of foods (original)
33.My child decides that s/he doesn't like a food, even without tasting it (original)
24.My child is difficult to please with meals (original)
Food responsiveness 1 
 28. Even if my child is full up s/he finds room to eat his/her favourite food (both this and original study)                          
 34. If given the chance, my child would always have food in his/her mouth(both this and original study)                          
 14. If allowed to, my child would eat too much (original)
Food responsiveness 2 
12. My child is always asking for food (both this and original study)
19. Given the choice, my child would eat most of the time (both this and original study)  
Drink desire 
6.My child is always asking for a drink (both this and original study)                                                                            
29.If given the chance, my child would drink continuously throughout the day (both this and original study)                          
31.If given the chance, my child would always be having a drink (both this and original study)	

	Emotional overeating 
2. My child eats more when worried (both study and original study)
13.My child eats more when annoyed (both this and original study) 15.My child eats more when anxious (both this and original study) 
27. My child eats more when s/he has nothing else to do (original)
Two missing factors (were not found in this study)
Enjoyment of food (EF)
1.My child loves food
3.My child has a big appetite
5.My child is interested in food
20. My child looks forward to mealtimes
22. My child enjoys eating
Satiety responsiveness (SR)
17. My child leaves food on his/her plate at the end of a meal
21. My child gets full before his/her meal is finished
26. My child gets full up easily
30. My child cannot eat a meal if s/he has had a snack just before

	
	
